# Four-dot Artifact on Automated Perimetry

**Published:** 2010-04

**Authors:** Mohammad-Reza Razeghinejad, Mohammad-Reza Khalili

**Affiliations:** Poostchi Ophthalmology Research Center, Shiraz University of Medical Sciences, Shiraz, Iran

A 74-year-old woman was referred to the glaucoma unit for pseudoexfoliative glaucoma. Upon presentation, visual acuity in both eyes was 20/30. Intraocular pressure was 18 mmHg in the right eye and 20 mmHg in the left eye with latanoprost eye drops. Ophthalmoscopy revealed a cup-disc ratio of 0.7 with superior neural rim notching in the right eye and concentric cup-disc ratio of 0.6 in the left eye. She underwent central 24-2 automated perimetry with the Humphrey Visual Field Analyzer (HVFA II, Carl Zeiss Meditec, Dublin, USA) using the SITA-fast program. Although she was instructed before the test, she was unable to respond to the early stimuli efficiently which was reflected in lower threshold values for initial test points. The reliability indices were within normal limits except for higher than normal fixation loss. On repeat visual field testing, reliability indices were within normal limits and thresholds of the four areas with poor sensitivity improved and the artifact disappeared (Fig. 1).

## DISCUSSION

Standard automated perimetry has been the gold standard for detecting and monitoring functional damage in glaucoma.^1^ In the interpretation of visual fields, artifacts should always be taken into account. In this report we present a type of artifact which we named the “four-dot artifact”.

In HVFA, thresholding is performed at four primary locations, 9 degrees apart from fixation in each quadrant. At these four points the stimuli are presented randomly before the test proceeds to other points.^1^ One common and well-known artifact is the clover-leaf defect in which the patient performs well at the beginning of the test but become inattentive with time and fails to respond to the stimuli.^1^ The result is a normal or near-normal central field with a dark periphery, resembling a clover-leaf. This may also be associated with a high false negative rate. In contrast, in the four-dot artifact, a reverse process happens. In other words, the patient does not respond to initial stimuli but then due to the learning effect, responds well to the following stimuli. In those who learn fast, the number of surrounding points with poor sensitivity is fewer as compared with those who learn at a lower rate. This artifact represents an intra-test learning effect; as the test progresses, the patient becomes familiar with the test strategy and responds appropriately. The learning effect further improves patient’s performance upon repeat testing, causing the artifact to disappear.^2^,^3^

Our patient showed a type of learning effect that occurred during the first test (intra-test learning effect) and all artifacts disappeared on the second visual field (inter-test learning effect). The mechanism underlying this artifact is exactly the opposite of the clover-leaf artifact. We defined this artifact based on the logic of the Humphrey visual field analyzer.

## Figures and Tables

**Figure 1:**
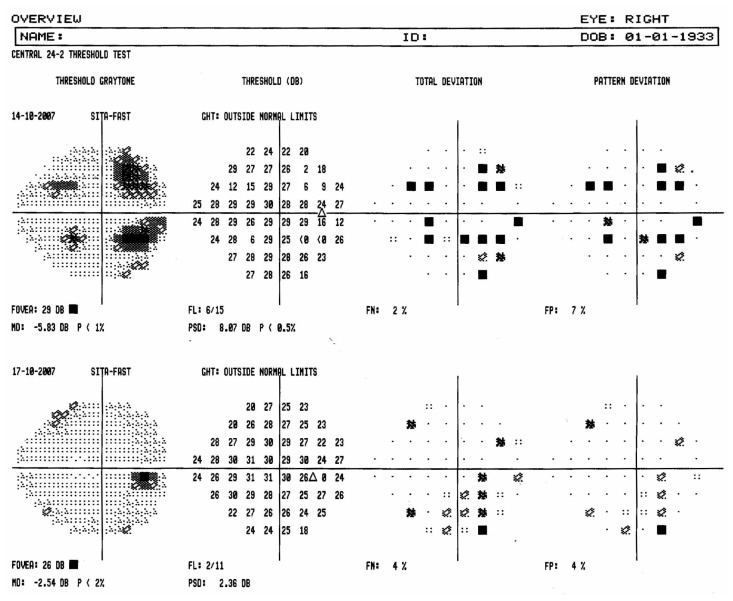
Overview printout of a patient with pseudoexfoliative glaucoma demonstrates the four-dot artifact which disappeared on test repetition.
